# Epigenetics, hypersensibility and asthma: what do we know so far?

**DOI:** 10.1016/j.clinsp.2023.100296

**Published:** 2023-12-03

**Authors:** Douglas da Silva Lima, Rahuany Velleda de Morais, Ciliana Rechenmacher, Mariana Bohns Michalowski, Marcelo Zubaran Goldani

**Affiliations:** a Programa de Pós-Graduação em Saúde da Criança e do Adolescente, Departamento de Pediatria, Universidade Federal do Rio Grande do Sul, Porto Alegre, RS, Brazil; b Laboratório de Pediatria Translacional, Centro de Pesquisa Experimental, Hospital de Clínicas de Porto Alegre, Porto Alegre, RS, Brazil; c Universidade Federal de Ciências da Saúde de Porto Alegre, Porto Alegre, RS, Brazil; d Serviço de Oncologia Pediátrica, Hospital de Clínicas de Porto Alegre, Porto Alegre, RS, Brazil; e Faculdade de Medicina, Universidade Federal do Rio Grande do Sul, Porto Alegre, RS, Brazil

**Keywords:** Asthma, Children, DNA methylation, Epigenetics

## Abstract

In this review, we describe recent advances in understanding the relationship between epigenetic changes, especially DNA methylation (DNAm), with hypersensitivity and respiratory disorders such as asthma in childhood. It is clearly described that epigenetic mechanisms can induce short to long-term changes in cells, tissues, and organs. Through the growing number of studies on the Origins of Health Development and Diseases, more and more data exist on how environmental and genomic aspects in early life can induce allergies and asthma. The lack of biomarkers, standardized assays, and access to more accessible tools for data collection and analysis are still a challenge for future studies. Through this review, the authors draw a panorama with the available information that can assist in the establishment of an epigenetic approach for the risk analysis of these pathologies.

## Highlights

• Epigenetics, hypersensibility and asthma: what do we know so far?
• Asthma can be triggered from epigenetic changes.
• Early life exposures can contribute to the development and exacerbation of asthma.
• DNA methylation profile of certain genes is related to asthma and allergy.
• Nasal epithelium samples have shown promise as a tissue for studying DNA methylation changes related to asthma.
• To find biomarkers and standardized assays to advance the field of epigenetics in respiratory disorders are still a challenge.

## Introduction

According to Cavalli and Heard (2019), epigenetic means “The study of molecules and mechanisms that can perpetuate alternative states of gene activity in the context of the same DNA sequence”. This perspective aims to cover transgenerational inheritance, mitotic inheritance, and the persistence of genetic activity or chromatin states for periods of time, even without cell division. It can also be related to environmental exposures leading to structural damage or functional changes in cells, tissues, and organs.^1^, ^2^, ^3^ In this context, the epigenome represents the interface between genes and the environment and provides a particular opportunity to recognize this intersection of causal components.^4^

The relationship between epigenetic changes and their results was first described by Conrad Waddington, in the 1940s, introducing the theory of “Epigenetic Landscape”.^5^ From there, numerous studies were developed and recent researchers were able to design how a single-cell unit would become a complex of differentiated cells, with specific functions, expressing specific sets of genes as a result of cellular identity mechanisms. Further, the authors are able to understand how phenotype becomes an aftermath of genotype and its interplay with the environment.^1^

The trajectory of Developmental Origins of Health and Disease (DOHaD) studies suggests a mesh with those causal components and epigenetic variants, considering that in-utero and post-natal life can induce biological changes leading to distinct outcomes.^3^

## Epigenetics and DNA methylation

The importance of epigenetic events is based on their long-lasting effects, starting from early life (gestation) until adulthood.^6^ Besides, epigenetic regulation is highly specific, may induce short and long-term changes in gene expression, and may be passed on from one generation to another.^7^ For this reason, they provide a baseline of the environmental influence on gene expression and disease hazard.^8^

**Fig. 1:**
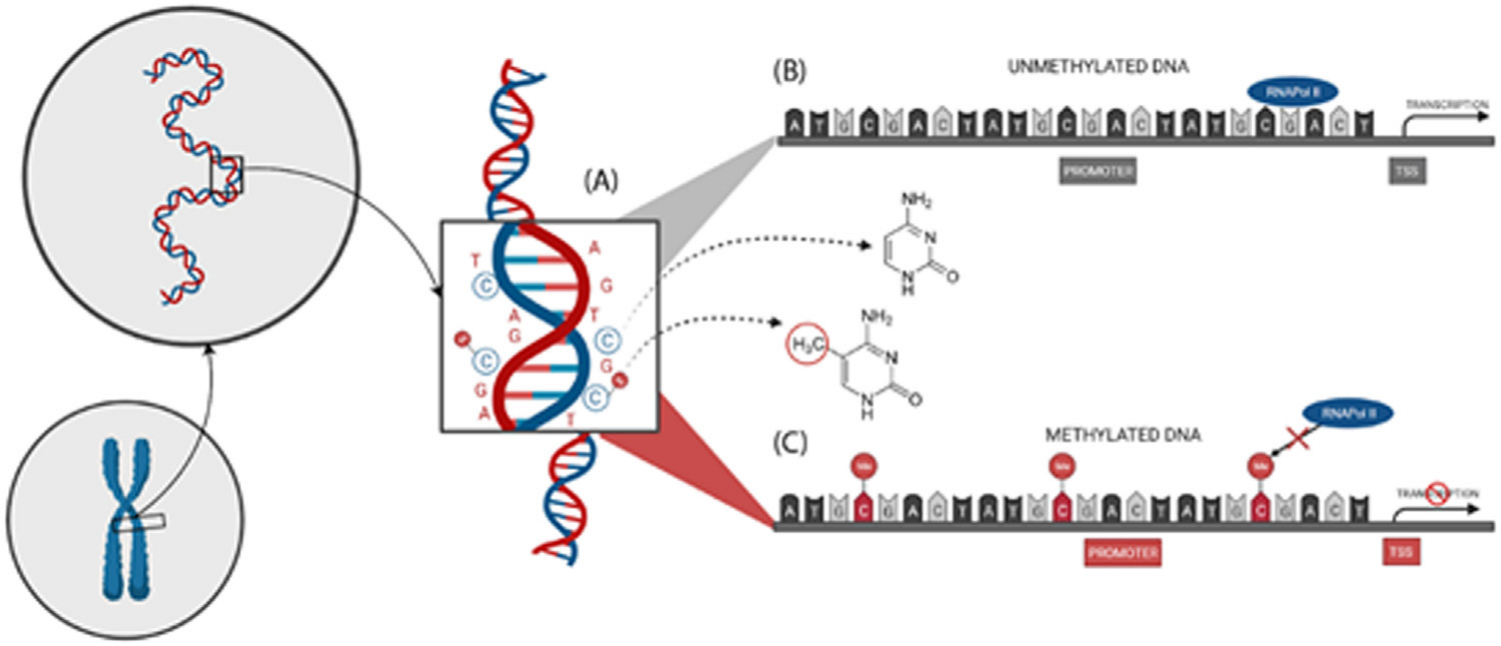
(A) A methyl group addition to the cytosine carbon 5 in cytosine-phosphate-guanine (CpG); (B) No CpG methylation in the gene's promoter region leads to active transcription; (C) Methylation in the promoter region of genes leads to transcriptional repression. TSS, Transcription Start Site; RNA pol II, RNA polymerase II.

Among possible epigenetic biomarkers, DNA methylation (DNAm) is likely the most prominent and frequent approach.^9^ DNAm consists of the addition of a methyl group onto the C5 position of the cytosine to form 5-methylcytosine, and is predominantly found at Cytosine-Phosphateguanine (CpG) dinucleotide sites.^4^^,^^10^ Initially, DNAm was proposed as the only carrier of epigenetic information, but later it was accepted that chromatin, proteins, and non-coding RNAs also act in this process. Likewise, variations in histones can influence the local structure of chromatin, directly or indirectly. Those variations can be inheritable, but reversible and may vary in different parts of the genome at different stages of the life cycle. Recent studies of cell reprogramming have shown that DNAm and chromatin can also behave as epigenetic barriers, preventing alterations in gene expression and cell identity. Nevertheless, chromatin states may impact transcriptional factor binding and DNA sequence polymorphism, targeting the balance of genomic mutability and stability.^1^^,^^3^ Altogether, those mechanisms compound epigenetic regulation that controls several processes, like cellular response to endogenous and exogenous stimuli, leading to healthy conditions or diseases (Fig. 1).^6^ As an example, cell differentiation in embryogenesis is triggered by DNAm.^5^

Human health is influenced by the exposure to the environment. In this perspective, the in-utero life must be imperatively considered, once exposures through this period may disturb the immune system, lung growth, and respiratory performance later in life.^6^ It has been shown that extrinsic factors during the perinatal period, such as adverse environment and maternal depression may impact the DNAm of the offspring and future outcomes inducing physical and psychiatric diseases. Otherwise, a good standard of nurturing may lead to healthier outcomes, inducing a “reshape” effect of DNAm during a lifetime.^3^^,^^10^ It is also important to highlight that genetic variants can explain how environmental factors impact epigenetic modifications.^2^ In this scenario, environmental exposures are likely to contribute to the increase in allergic diseases.^11^

## Epigenetic paths for hipersensibilization

Allergic diseases are increasing on a global scale. In the United States, US$ 18 billion is spent annually on their treatment.^12^ As previously mentioned, the early years of life play a decisive role in atopic diseases.^9^

Methylation patterns and gene expression changes are well connected to persistent atopic asthma in respiratory epithelia of American children and some genes are significantly correlated to immune response.^13^ In a recent trial by Popovic et al., 2019, a case-control study nested in a birth cohort, with 136 children between 6 and 18 months old, to figure the association of early childhood wheezing and DNAm, has observed a longitudinal temporal tendency towards allergic sensitization, reinforcing the idea that the methylation profile at birth can be a marker of allergic vulnerability during childhood.^3^ DNAm is known to be associated with asthma, which can be triggered by smoking, air pollution, indoor contaminants, and allergens. Passive tobacco exposure decreases lung function and increases the risk of respiratory diseases. Also, prenatal tobacco smoking exposure increases the risk for the development of asthma in the neonate.^14^ In a similar manner, air pollutants or allergens can go through the airway epithelial barriers and reshape immune responses.^15^ Likewise, air pollution as well as viral infections and intestinal and pulmonary dysbiosis can induce sensitization, exacerbations, or trigger the onset of asthma.^6^

In 2018, Yang et al. used nasal epithelium samples from African-American children with allergic asthma to compare methylation profiles of genomic DNA. They were able to identify 186 genes with methylation changes, including genes related to atopy, asthma, immunity, airflow obstruction, and epigenetic regulation. By showing that the epigenetic marks on the respiratory epithelium are related to allergic asthma, this study provided a basis for studies of nasal DNA in larger populations.^16^ In another study, DeVries et al. suggest that childhood asthma engages epigenetic alterations in immunoregulatory and pro-inflammatory pathways.^17^ Thus, asthma has an epigenetic regulatory mechanism influenced by genetic variability and environmental exposures, that can be used as a strong biomarker.^12^

An important issue in these studies is the origin of the tissue being analyzed. Most allergic biomarkers to date were extracted from whole blood DNA. Also, cord blood immune cells were adopted to establish (in an effort to predict) DNAm signatures related to allergy and asthma.^17^ Although manifested at birth, these epigenetic signatures are still able to change, as determined by physiological pathways. It means that some gene alterations might be transient, or disease-related. Therefore, DNAm mechanisms may present some malleability during lifespan. Besides, most indicators of allergic disease and asthma are related to epigenetic aging of cells collected from nasal epithelia.^4^ Data on the differences and changes in the methylation profiles of these different tissues and their implication in hypersensitivity reactions are still very few.

## Asthma: an epigenetic outcome

Asthma is one of the most common pulmonary chronic diseases. It may start early in life and, if not handled properly, tends to cause loss of pulmonary function in adult life.^18^ It is characterized by reversible airflow obstruction and airway inflammation with symptoms such as wheezing, shortness of breath, cough, and chest tightness, that may vary over time and frequency, intermittent or persistently. Some of them are transient and tend to disappear at school age. Other persistent symptoms are up to remain until adulthood.^11^ The onset and clinical progression are strongly connected to environmental exposures and genetic susceptibility.^4^^,^^8^ Environmental and genomic aspects as well as aberrant immune maturation early in life may engage the disease outbreak.^19^

In the past decade, the increase in morbidity and mortality related to respiratory diseases has reached the spotlight in several countries.^13^ Epidemiological evidence has shown that approximately 30% of children will experience at least one episode of wheezing in their lifetime.^2^ Worldwide, asthma is frequently diagnosed during pre-scholar years and affects more than 300 million people.^20^ The estimated heritability of the disease in certain studies starts from 40% to 60%^4^^,^^8^ and can reach as high as 80%.^21^ However, the timeline and mechanisms of asthma onset are not well described yet.^3^^,^^22^

To keep homeostasis in human lungs, a healthy adaptation to the environment is needed and the lung epithelia must present a competent mucosal immunity. The respiratory system provides mucociliary clearance and a barrier against pathogens and other particles that may affect the alveolar gas exchange. An unbalanced immune system or deficient epithelial barrier can therefore result in respiratory illness, as the respiratory system remains in unprotected contact with exogenous factors.^4^^,^^14^

Protection factors are also described. Some exposures can promote healthier airway development even in the presence of a genetic predisposition to the disease. Recent studies propose that the use of fish oil and vitamin D throughout pregnancy may minimize the risk of early childhood wheezing. Also, children from a farm environment who are frequently exposed to allergens, bacteria, fungi, and others from diverse microbiomes and the consumption of unprocessed cow milk may develop a strong protective barrier against asthma and allergy. Indeed, children from rural areas have shown a prevalence of allergic diseases lower than children from urban environments.^6^^,^^19^

Asthma management is based on the severity and risk profile of these children. Many recent studies have evaluated the relationship of genetic polymorphisms as potential risk factors for the onset and severity of asthma symptoms.^7^^,^^11^^,^^19^ Even with the advance of asthma treatment, there is still a group of patients that faces a low response to traditional approaches. From this, a better understanding of asthma and allergy mechanisms through the observation of DNAm and gene expression may provide a clue about reliable biomarkers associated with therapeutic responses in this population.^13^

There is a strong connection between asthma and DNAm^3^^,^^4^^,^^23^ (Table 1). In 2019, Reese et al. conducted a study with newborns and school-age children. They evaluated the entire epigenome by observing the presence of CpG methylation in the blood of these two age groups both in prospective and cross-sectional analyses. In 8 cohorts of newborns (668 cases), 9 CpGs islands and 35 regions were differentially methylated in patients who developed asthma, while in older children, in a cross-sectional analysis, 179 CpGs islands and 36 differentially methylated regions were identified in these cases.

**Table 1:** Characteristics of DNAm studies on asthma demonstrating heterogeneous techniques applied among the trials.

Author	Patients	Sample	Main Results
Reese et al., 2019	n = 1299 Age = 7-17 years old	Whole blood	Identified epigenetic variations related to asthma in newborns and children.
Cardenas et al., 2019	n = 1083 Age = 12 to 65 years old	Nasal swab cells	285 CpGs sites associated with asthma.
Arathimos et al., 2017	n = 1529 Age = 7.5 years and 16.5years	Peripheral blood	*IL5RA* and *AP2A2* gene methylation related to asthma at 16.5 years old.
Popovic et al., 2019	n = 136 Age = 6 to 18 months	Saliva	*PM20D1* gene hypermethylation associated with early childhood wheezing.
Yang et al., 2018	n = 78. Age = 10 to 12 years old	Nasal epithelia	186 genes related to atopy, asthma, immunity, airflow obstruction and epigenetic regulation.
Ning et al., 201934	n = 182 children. 3 to 14 years old	Peripheral blood	*ADAM33* polymorphism is correlated with increased susceptibility to asthma.
Nicodemus- Johnson et al., 2016	n = 115 adults. 26 to 52 years old	Airway epithelial cells	Regulatory locus associated with asthma risk and epigenetic signatures of specific asthma endotypes.

Another interesting fact is that DNAm is tissue-specific. In the case of diseases whose local aspects are pronounced, such as asthma, the selection of tissues to carry out the DNA methylation profile can be an important variable in the analysis.^3^ DNA methylation of the nasal epithelium may offer more information about changes in the airways, once whole blood DNA does not perform a trustworthy complete agreement for pulmonary sample studies.^24^ Cardenas et al. (2019) conducted an Epigenome-Wide Association Study (EWAS) with nasal samples of 547 children to analyze current asthma, allergic sensitization, and rhinitis, Fractional exhaled Nitric Oxide (FeNO), and lung function, where diverse differentially methylated CpG's and regions were found related to these diseases, suggesting that the nasal cellular epigenome may be a good biomarker to airway inflammation in children. Also, in peripheral blood samples, it is crucial to consider confounding factors, such as leukocytes and eosinophils, that may lead to biased results.^2^^,^^4^

Some data contradict the tissue specificity theory in the analysis of patients with asthma. A recent study by Vieira Braga et al. (2019) was designed to find differences between asthmatic and healthy patients. A wide overlapping of the cellular epigenetic profile of the different locations of the respiratory tree was observed. In fact, a diversity of immune cells was found in different parts of the respiratory unit. Therefore, it could be observed that, in asthmatic patients, airway wall remodeling was highly correlated with the presence of a chronic inflammatory response, which may cause a cell-to-cell signaling network.^25^

Also, variations in innate and adaptive immune responses appear to precede the diagnosis of asthma in children. This group of respiratory and immune modifications may characterize a window of susceptibility toward the disease and can contribute to asthma pathogenesis. The disease trajectory can also be affected by post-translational histone modification that influences genetic regulation and its possible outcomes.^23^

## Genes in sight

Several genes whose altered DNA methylation has been described in asthmatic individuals have different pathophysiological aspects and relationships with components and functions of the immune system.^26^ Some of them deserve special attention (Table 1).

### IL5RA

The *IL5RA* gene (Interleukin 5 Receptor Alpha subunit) is found on the human chromosome 3. Hypomethylation of the *IL5RA* gene in Airway Epithelial Cells (AECs) and eosinophils has been shown to be a potential therapeutic target for asthma. In an EWAS, Arathimos et al. (2017) used data from the Avon Longitudinal Study of Parents and Children (ALSPAC), with more than 1,500 patients, identifying 302 CpGs related to asthma and 405 to wheezing, with a robust overlap between them.^2^ After adjusting for cell count, there was a difference of 2.49 (95% CI -1.56, -3.43) percent of methylation in patients with asthma. The *IL5RA* gene also showed an important link with the pathogenesis of asthma in a multi-omics approach.^21^ The hypomethylation of *IL5RA* gene on airway epithelial cells and eosinophils has shown to be a therapeutic target for asthma. The epigenetic finding allowed the study and incorporation of the monoclonal antibody Benralizumab^21^ (Anti-IL-5Ra) in clinical practice, a drug that binds to *IL5RA*, inducing a reduction in exacerbations and improvement in lung function in patients with severe asthma.^4^^,^^6^^,^^21^^,^^27^

### EPX

Also common in eosinophils and AECs, the *EPX* (Eosinophil Peroxidase) gene, is involved in granulocyte functions, notably neutrophils, cytokine production and signaling.^26^ The enzyme expressed from eosinophil peroxidase is released to protect from parasitic infection and allergic stimuli.^2^ The gene is found clustered with other peroxidase genes on chromosome 17. Lower DNAm of the *EPX* is related to allergic asthma. In a study with 483 Puerto Rican children and adolescents, the methylome from nasal epithelial cells was studied. It was observed that 61% (48/79 CpGs), including multiple CpGs annotated to EVL and *EPX* genes, were associated with asthma outcomes, such as allergic asthma, environmental IgE sensitization, FeNO, and total IgE (FDR < 0.05).^4^^,^^16^^,^^24^

### SMAD3

The *SMAD3* (*SMAD Family Member* 3) is a protein-coding gene located at chromosome 20p13. It plays an important role in immune response regulation and acts together with other proteins to promote fibrosis regulation.^23^^,^^28^ The hypermethylation of the *SMAD3* gene promoter is associated with asthma, particularly in children of asthmatic mothers. A study by DeVries et al. (2017) has shown that children from asthmatic mothers had *SMAD3* gene methylated at birth. The methylation profile was analyzed from cord blood samples of children from three different cohorts. Methylation levels measured by bisulfite sequencing over the entire DNA (Spearman correlation coefficient [ρ = 0.48], p = 1.2 × 10−13) and at intermediate DNA methylation levels (8%–92%, p = 0.006), *SMAD3* methylation was extremely correlated with asthma (ρ = 0.46, p = 0.009).^22^

## Future perspectives

Studies on epigenetics have advanced, but despite this, it is not yet clear whether changes in these pathways have a direct causal relationship in certain diseases. For this reason, it is essential to better understand the connection between the pathogenesis of pathologies, and their environmental and developmental influences. More systematic studies are needed to detect how DNAm paves the way for certain diseases.^22^ However, integrative analysis with potent causal inference and longitudinal data is still a challenge.^6^

A relevant point in carrying out these analyses is the origin of the biological material used for the study. Although many surveys use blood, samples from other locations can also be used. Nasal epithelium, for example, has often been used to study airway diseases, including cystic fibrosis, allergic sensitization, asthma, and other obstructive respiratory disorders.^24^ Nasal epithelia is easier to access than blood samples and may be a profitable proxy for pathological changes in lung cells.^6^^,^^29^

In addition to the origin of the biological material used, the association between environmental exposure and epigenetic variations is another challenge. The ability to compare two or more samples at different time points with the same individuals in the cohort can ensure a strong approach to data analysis.^2^ Genetic predisposition, epigenetics, and exposure are perfectly suited to be studied through birth cohorts. Currently, the influence of intergenerational events on epigenetic mechanisms that can induce changes in the health-disease pattern is also evidenced. Studies indicate that the health of offspring is influenced by intergenerational aspects related to maternal and grandparent's exposures and health status.^9^

The development of an “asthma phenotype index”, considering molecular and clinical criteria, with predictive values of management would be relevant for asthma diagnosis and modulated treatment.^30^ On the other hand, the small number of studies available today apply different designs and analysis techniques. This fact precludes robust conclusions about DNAm and the epigenetic mechanisms of asthma.^24^ Machine learning analytics can provide substantial benefits in analyzing this heterogeneity, but little data is available to allow the use of this technology.^31^ The characterization of epigenetic alterations with a homogeneous approach and standardized techniques for disease outbreak and progress remains needed.^8^ New studies in the areas of genomics, biochemistry, and genetics can facilitate the understanding of how epigenetic mechanisms influence the evolution of these patients, promoting safer and more economical clinical approaches.^1^ In addition, these studies may seek to define how many aspects of life can influence the onset of inflammatory pathologies and asthma. Epigenetics studies fit a new perspective in understanding pulmonary diseases. It is possible that methylation profile analyses may soon be part of the diagnosis and risk stratification for childhood asthma.

## Conflicts of interest

The authors declare no conflicts of interest.
